# Wildfires, smog, and the persistent threat of air pollution to human health

**DOI:** 10.1016/j.ebiom.2025.105602

**Published:** 2025-02-12

**Authors:** eBioMedicine

2025 began with the devastating news of widespread fires in Los Angeles (CA, USA), with the loss of homes, possessions, and human life. Eventually, these fires will be extinguished and the immediate danger will subside, but their release of smoke and fine particulate matter into the atmosphere as air pollution highlights a substantial lingering human health hazard.

A health impact assessment study quantifying the number of global deaths attributable to air pollution from landscape fires published in *The Lancet* in December, 2024, put the figure at 1.53 million all-cause deaths per year, with particulate matter smaller than 2.5 μm (PM_2.5_) contributing to 77.6% of these deaths. Wildfires themselves are a natural part of many ecosystems and not all are a direct consequence of human activity. However, their magnitude and frequency, fuelled by widespread and persistent drought conditions, is increasing. During 2023–24, there were record-breaking wildfires in Canada, Australia, Brazil, and many other regions of South America. Although *The Lancet* health impact assessment study incorporated data from 2000 to 19, the human health consequences of several of the largest fires of the 21st century, having occurred since 2020, remain unevaluated.

Wildfires are intermittent, but anthropogenic PM_2.5_, generated through human activity, is also produced continuously by the burning of fossil fuels for energy and transportation and through the wearing down of tyres and brakes. As such, high levels are found in densely populated cities, such as Delhi, Dhaka, and Lahore, where weather conditions, landscape topology, and air pollution combine to form dangerous smog events. Much like smoking, long-term exposure to PM_2.5_ is a risk factor for lung cancer, and even short-term exposures are now understood to have detrimental effects on pulmonary health.

Two nationwide studies on the short-term effects of air pollution on mortality in people with chronic pulmonary diseases in China have been published in *eBioMedicine*. In December, 2024, research investigating the 2.26 million deaths from chronic obstructive pulmonary disorder in China during 2013–19 established that increased PM_2.5_ was associated with increased mortality. In September, 2024, a similar study of 19,320 deaths from bronchiectasis, a chronic lung disease with different causes and treatments, found a similar association across the same time period. In both studies, researchers found that PM_2.5_ concentrations were linearly correlated with risk of death, and that increased mortality in these populations was evident as early as the very first day of PM_2.5_ exposure. These health risks are not geographically restricted, however, as shown by a large meta-analysis of 33 studies across North America, Europe, Oceania, and Asia. This research, published in *eBioMedicine* in December, 2022, highlighted an increased risk of cardiovascular morbidity worldwide within just 3 h of exposure to elevated PM_2.5_.

Despite these data, the mechanisms by which air pollution translocates into human tissue in real-world conditions remain poorly understood. In *eBioMedicine* in December, 2024, Van Pee and colleagues used femtosecond pulsed laser illumination, a technique previously developed by the same group, to show that fine black carbon particles accumulate in the intestinal submucosa, a highly vascularised tissue. This study presents an additional plausible mechanism, beyond the alveolar surfaces in the lung, by which air pollution might enter the bloodstream and exert detrimental effects across the body. On this topic, this group has also investigated the effect of maternal air-pollution exposure on kidney function in newborns. In Belgium, where these studies were conducted, air pollution is considered relatively low (ie, 10.8 μg/m³ PM_2.5_), yet this figure still surpasses the air quality standard levels set by the EU (ie, 10.0 μg/m³ PM_2.5_) and WHO (5.0 μg/m³ PM_2.5_), which aim to limit the effects of PM_2.5_ on human health.

So, what can be done to reduce exposure to air pollution? At the global level, efforts to limit climate change and resulting wildfires should be prioritised. Nonetheless, on Jan 20, 2025, via an executive order, the USA withdrew from the Paris Agreement for a second time, which aims to hold nations accountable for environmental promises made. However, local interventions continue to be influential; on Jan 5, 2025, New York City (NY, USA) became the first city in the USA to implement a congestion charge for vehicular traffic, joining many cities with similar schemes worldwide. A systematic review published in *The Lancet Public Health* in July, 2023, suggested that low-emission zone systems such as these can measurably alleviate the effects of air pollution on the health of people that live in cities.

However, more research is required to establish how the design of these systems can be optimised to promote health, and more needs to be done to ensure that income-based disparities of air pollution exposure and outcomes are not widened. In their Personal View published in *eBioMedicine* in July, 2023, Vilcassim and Thurston highlight that “the burden of air pollution is not equally shared”. Within cities, air pollution is highest in the most economically deprived areas. Similarly, *The Lancet* health impact assessment study stated that more than 90% of all wildfire air pollution-related deaths were in low-income and middle-income countries.

In the UK, 2024 saw both the closure of the last coal-burning powerplant and record electricity generation through wind power. There is hope that energy transitions such as these will continue to reduce the production of air pollution, at least of that which originates through deliberate, and avoidable, human activity.

A deeper understanding of the human health consequences of airborne pollutants, as well as the biological mechanisms that drive them, is crucial to evaluating and restricting their impact. Key questions remain in how the chemical constituents of PM_2.5_ differ in their immunotoxicity and, although air pollution during pregnancy is known to be associated with changes to fetal growth and neurodevelopment, the specific molecular mechanisms by which these particles exert epigenetic control are largely unknown. *eBioMedicine* welcomes research into precision environmental health and prospective exposure monitoring to further understand the effects of air pollution on human health and disease.

